# Physical and Mechanobiological Basis of Biological Functions of Platelets

**DOI:** 10.1055/a-2676-4451

**Published:** 2025-08-20

**Authors:** Shinya Goto, Shinichi Goto

**Affiliations:** 1 Department of Medicine (Cardiology), Tokai University School of Medicine, Isehara, Japan; 2 Division of Cardiovascular Medicine, Brigham and Women's Hospital, Boston, Massachusetts, United States; 3 Harvard Medical School, Boston, Massachusetts, United States

**Keywords:** platelets, adhesion, von Willebrand factor, GPIbα, GPIIb/IIIa, molecular dynamic simulation, high-performance computer

## Abstract

Platelets play a unique role in thrombosis and hemostasis. Historical research has revealed biological mechanisms underlying various platelet functions. However, unraveling the complex mechanisms underlying various platelet functions is challenging. Recent progress in high-performance computer has enabled an understanding of the complex biological functions of platelets through combinations of basic principles of physics, such as Newton's laws of motion, fluid mechanics, and mechanobiology. Platelets are blood cells with diameters of 2 to 5 µm. They lack nuclei but contain organelles such as mitochondria. Platelets promptly adhere to the sites of endothelial damage for hemostasis. Adherent platelets are activated to allow plasma ligands of fibrinogen and von Willebrand factor (VWF) to bind stably to them. They also enhance local coagulant activity through their procoagulant activity. The specific biological functions of platelets are mediated by dynamic structural changes in their membrane proteins. Even lipids and proteins that mediate the specific functions of platelets are constructed from atoms following basic physical rules, such as Newton's laws of motion. Thus, the various biological functions of platelets can be constructed from physical principles, starting with the movement of atoms. Here, various complex biological functions of platelets were constructed using mathematical models and simple physical principles. This framework may help explain the complex pathophysiological mechanisms underlying the VWF–platelet interaction in both healthy and diseased conditions. Detailed quantitative biological experiments confirmed the validity of these mathematical models. The future direction of constructive “theoretical medicine and biology,” starting from atomic movements, is expected to follow.

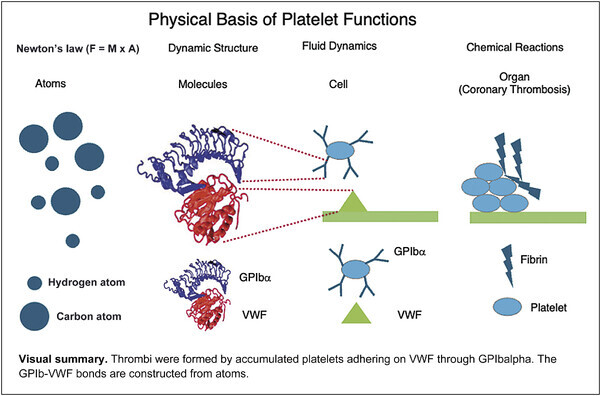

## Biological Role of Platelets

Platelets are small cells without nuclei and do not have the potential to differentiate or divide; however, they contain other subcellular organs, including the mitochondria and Golgi apparatus. 1 The shape of non-activated platelets is discoid with a diameter of 2 to 5 µm. 2 However, it changes substantially after activation. 3 The concentration of platelets in humans is approximately 200 × 10 ^3^ /µL, and the total volume of platelets in circulating blood is small compared to the other blood cell types, including erythrocytes. Platelets play crucial roles in hemostasis and thrombus formation. 4 5 6

Recent progress in biological research has revealed that the biological functions of platelets extend beyond hemostasis or thrombus formation, 7 8 9 including regulating inflammation, 10 11 12 various immunological functions, 13 14 15 16 17 18 and maintaining microcirculations. 19 20 Recent progress in single-molecule biophysics has further revealed the biomechanical behavior of platelets. 8 9 The physical basis of the biological functions of platelets will be discussed in this review.

## Physical Basis of Platelet Adhesion

**Fig. 1:**
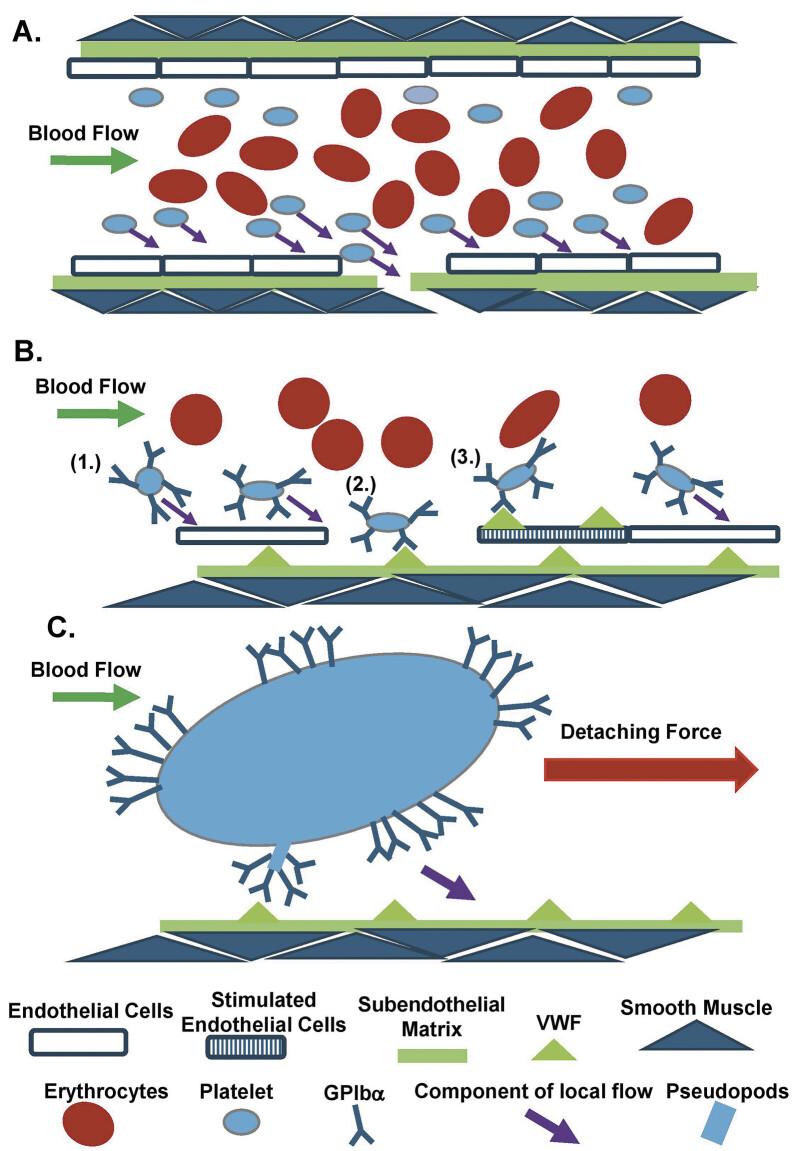
Platelet Adhesion at Sites of Endothelial Damage Under Blood Flow Conditions. ( **A** ) Heterogenous distributions of blood cells in flowing blood. Owing to the effects of blood flow, the distribution of circulating blood cells is inhomogeneous. Red cells tend to stay in the center of blood flow due to Fåhræu effects. 23 Then platelets are pushed in the direction of the vessel wall by erythrocytes, as shown by the purple arrows. ( **B** ) Mechanism of platelet adhesion to the sites with endothelial injury. The vessel wall is covered by endothelial cells that prevent platelet interaction with the subendothelial matrix. (1) Of the subendothelial matrix, the von Willebrand factor (VWF) plays a crucial role in capturing platelets flowing to the vessel wall. Platelets express the VWF receptor glycoprotein (GP) Ibα in a constitutively active conformation. Once endothelial cells are disrupted by physical (2) or biochemical stimulation (3), platelet adhesion begins promptly upon exposure of VWF to the bloodstream. Flowing platelets possessing flow vectors toward the vessel wall, as shown by the purple arrows, enable platelets to adhere to the site of endothelial injury through simple physical phenomena. **(C)** Mechanism of single platelet adhesion on VWF exposed at the sites of endothelial injuries. VWF molecules have multimeric structures. Platelet GPIbα molecules are heterogeneously expressed. Most likely, the GPIbα molecules that are densely expressed on flowing platelets transiently bind to a single subunit of VWF expressed on the vessel wall. When the number of bonds formed between VWF and GPIbα becomes sufficiently large to produce a binding force strong enough to resist the fluid dynamic detaching force, the platelets apparently adhere to the site of the injured vessel wall. The fluid dynamic forces applied to the platelets and heterogeneous cytoskeletal tension result in the elongation of the platelets, forming pseudopods (shown as blue rectangles) to support their binding to the vessel wall. Notably, the GPIbα–VWF bond was always transient, with a high off-rate.

Biological reactions, including platelet activation, require more time than simple physical phenomena. Hemostatic reactions begin immediately to avoid the loss of blood components when blood vessels are injured. Accordingly, simple physical reactions are more suitable than complicated biological mechanisms for rapid hemostasis. Indeed, biological experiments have revealed that platelet adhesion occurs at sites of endothelial damage with almost no delay. 21 22 The prompt platelet adhesion is most likely mediated by simple physical reactions rather than biological mechanisms. Supporting this idea, experiments have shown that platelets tend to flow adjacent to the vessel wall owing to the axial accumulation of erythrocytes ( Fig. 1A ). The large and heavy erythrocytes accumulate at the center of blood flow by Fåhræus's effect in microcirculation. 23 24 This axis concentration of erythrocytes has been known since the 18th century. 25 Platelets are smaller and lighter than erythrocytes. They are pushed toward the vessel wall by the fluctuating movement of erythrocytes. These biological findings were supported theoretically by computer simulation calculations based on fluid mechanics. 26 27 The validity of the calculation results of hematocrit-dependent increase in the rate of platelet adhesion was confirmed by quantitative biological experiments using human blood and a rectangular flow chamber with statistical correlation analysis. 27

Owing to the effects of the axis concentration of erythrocytes, platelets are pushed toward the blood vessel wall and collide regularly with it. However, platelets do not adhere to vessel walls in the presence of healthy functioning endothelial cells. In other words, endothelial cells protect vessel walls from platelet adhesion. 28 Once endothelial cell function is impaired, platelet adhesion begins. 21 22 These phenomena support the mechanism of platelet adhesion as summarized in Fig. 1B . Flowing platelets have a velocity vector toward the vessel wall generated by positional fluctuations of heavy erythrocytes flowing in the center of the blood flow. 26 27 Platelets express receptors for von Willebrand factor (VWF), namely, glycoprotein (GP) Ibα, in a conformation ready to bind with VWF. 29 The healthy endothelial cells cover VWF to prevent platelet adhesion to the vessel wall. 21 30 All these important premises for the mechanism summarized in Fig. 1B have been proven biologically.

VWF is an important ligand for platelet adhesion and cohesion under blood flow. 31 It is produced constitutively from endothelial cells 32 or stored in either Weibel-Palade bodies in endothelial cells or α-granules in platelets or megakaryocytes. 33 34 These stored VWFs are released upon stimulation. In endothelial cells, the prompt extracellular translocation of VWF occurs after stimulation. 22 35 36 Platelet adhesion to stimulated endothelial cells follows simultaneously. 21 22 When endothelial cell disruption occurs, VWF accumulated in the subendothelial matrix is exposed to the bloodstream with other thrombogenic substances such as collagen. 30 32 37 38 39 40 Once VWF is expressed at the vessel wall, platelet adhesion occurs promptly mediated by VWF binding with GPIbα. 1 A previous study suggested that platelet adhesion to the vessel wall can be simulated quantitatively using a kinetic Monte Carlo lattice model. Three types of events, including GPIbα diffusion, GPIbα–VWF bond formation, and their breakage, were included in their model. Simulation calculations suggested the importance of GPIbα localization compared to the probability of GPIbα–VWF bond formation in the adhesion force generated between the platelet and the vessel wall. 41

Single platelet cells express approximately 15,000 molecules of GPIbα. 29 It is important to note that the distribution of GPIbα expression on single platelets is heterogeneous, meaning that the GPIbα expression is concentrated in some regions of the platelet membrane. 1 Molecular dynamic simulation calculations and biological validation experiments revealed that a single bond of GPIbα–VWF generates binding forces of 50 to 100 pN. 42 43 44 Because platelets adhered on VWF at a wall shear rate of 1,500 s ^−1^ are exposed to a detaching force close to a few hundred pN, 45 only a few molecules out of the 15,000 GPIbα bound with VWF are necessary and sufficient for platelet adhesion to the vessel wall resistant to the detaching force generated by blood flow. The physical mechanism of single platelet binding to the vessel wall is summarized in Fig. 1C . In summary, the membrane expression of GPIbα is heterogeneous, and only a few GPIbα molecules bind to VWF at the nanometer scale, supporting single-micrometer-scale platelet adhesion at sites of the injured vessel wall. Because platelets start to adhere to the vessel wall in the region where GPIbα molecules are densely expressed, platelet adhesion is often supported by pseudopods formed by the elongation of the platelet membrane due to the effects of fluid dynamic force and the heterogeneity of cytoskeletal tensions as mechanobiological responses 46 ( Fig. 1C ).

## Physical Basis of Platelet Adhesive Protein Functions

The biological functions of various macromolecules, such as platelet GPIbα, are complex. Indeed, GPIbα is the main player in platelet adhesion at sites of endothelial damage under blood flow conditions. 31 47 Even though apparently complicated, the biological functions of various macromolecules should depend on the simple physical characteristics of the atoms constructing them. In simplified physics, atomic characteristics can be described by three parameters: (1) mass, (2) position coordinates, and (3) velocity. Accordingly, the physical characteristics of molecules can be described by integrating the positional coordinates and velocity vectors of atoms of various masses. Recent progress in high-performance computers has enabled the calculation of such parameters for all atoms constituting macromolecules with specific biological functions using a simple physical law, Newton's Second Law of F (force) = M (mass) × A (acceleration). Each atom in a molecule has its own initial position coordinate. Owing to heat and interactions with other atoms, each atom faces various forces, including the van der Waals force, Coulomb force, and universal gravitation. Around the target molecule, the force fields in which the forces act on the target atoms are uniquely determined by their relative positions. Among the various force fields, the CHARMM (Chemistry at HARvard Macromolecular Mechanic) force field, where quantum mechanics is incorporated into the Newtonian force field as a rotating and progressing spring, is popularly used. 48 49 50 51 52 The forces on all the atoms constructing the molecules are determined by the CHARMM force field. Then the position coordinates and velocity vectors of all the atoms constructing the molecule were updated in a stepwise manner following the equation F (force) = M (mass) × A (acceleration). Typically, small changes in the position coordinate and velocity vectors of each atom are calculated every 2 × 10 ^−15^ sec. Through integrated calculations, the physical parameters of platelet GPIbα binding with VWF, including binding structures, energies, and forces, can be calculated. 42 53

**Fig. 2:**
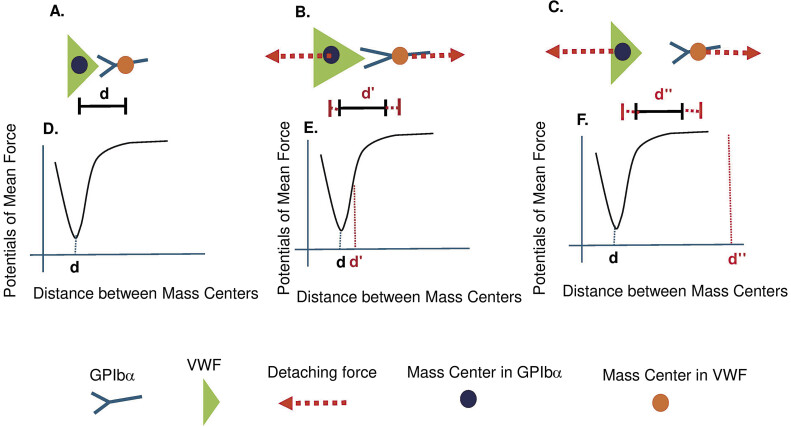
Physical Characteristics of GPIbα–Von Willebrand Factor (VWF) Bond. The structural energy expressed as the potential of mean force (PMF) in the GPIbα–VWF bond becomes the lowest in the absence of an external force when the distance of the mass of each molecule is d. ( **A** ) When the detaching force is applied to the mass centers of GPIbα and VWF in the direction of the orange dotted lines, the shapes of GPIbα and VWF are influenced by the effects of these external forces. The PMF of the GPIbα–VWF bonds increased with increasing distance between the mass centers of GPIbα and VWF, relative to d' ( **B** ). When the distances between the molecules reach the threshold (d''), they no longer interact with each other and detach ( **C** ). ( **D–F** ) The relationship between the PFM of the GPIbα–VWF bonds and the mass distances between GPIbα and VWF.

The calculation of the force generated between GPIbα and VWF should be conducted under the strict conditions shown in Fig. 2 . Fig. 2A shows the positions of GPIbα and VWF with a mass distance of d. The potential of mean force (PMF) between GPIbα binding and VWF (which can then be used to calculate the binding energy) becomes lowest at a mass distance of d. Then the PMF was calculated when the mass centers of GPIbα and VWF were gradually separated in each by 0.5Å. Finally, the distance between the two molecules becomes sufficiently large such that physical interactions are no longer present ( Fig. 2C ). Based on the calculated relationship between the mass center distance between GPIbα and VWF and the corresponding PMF, the binding force between GPIbα and VWF was 63.4 pN. 42 This value may not be the same as the binding force generated between GPIbα and VWF when platelet adhesion to VWF occurs under blood flow conditions. However, this value is still meaningful because it reflects the potential binding energy between the GPIbα and VWF bonds, and the values can be validated biochemically using other experimental methods. The predicted binding force (63.4 pN) is within the order of magnitude of the values experimentally measured with an optical tweeter and an atomic force microscope. 43 44

Because platelets adhered to the site of endothelial injury face a fluid dynamic force of approximately 100 pN at a wall shear rate of 1,500 s ^−1^ , several pairs of GPIbα–VWF bonds should produce a sufficient binding force to resist detachment from the vessel wall due to the detaching force generated by blood flow ( Fig. 1C ). 27 It is important to note that the GPIbα–VWF bond is not stable but only transient. 47 Accordingly, the number of GPIbα–VWF bonds formed at any given moment is probability-dependent. The actual behavior of platelets binding to VWF under flow conditions could be predicted using a multiscale model for shear-mediated platelet adhesion dynamics, which integrated dissipative particle dynamics (DPD) and coarse-grained molecular dynamics (CGMD) to describe molecular-scale intraplatelet constituents and their interactions with the surrounding flow using lattice kinetic Monte Carlo methods. 54

## Physical Basis of the Effects of Blood Flow on Platelet Adhesion

**Fig. 3:**
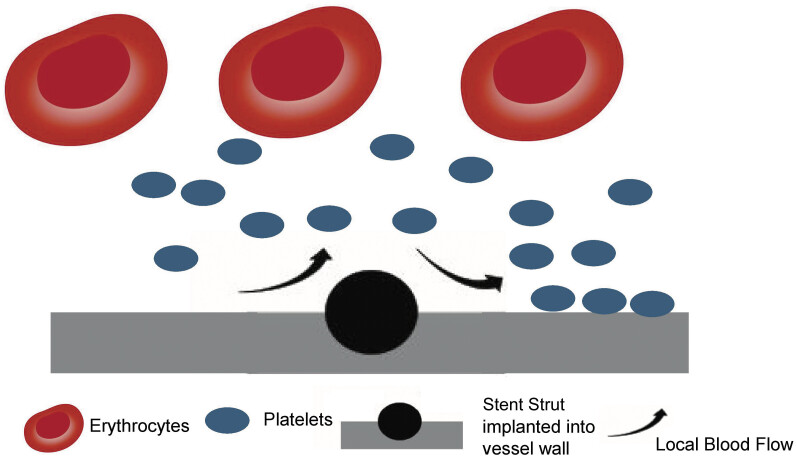
Platelet Accumulation Around Stent Struts. Stent struts were implanted into the vessel walls. Parts of the struts appear in the flow routes and disturb the local blood flow. Owing to the axial accumulation, erythrocytes flow at the center of the blood flow. Platelet flow adjacent to the vessel wall owing to the effects of the stent struts; platelets flowing adjacent to the vessel wall were pushed toward the center of the blood flow around the stent struts. Subsequently, the platelets moving toward the center of the blood flow are pushed back in the direction of the vessel wall by the effects of heavy erythrocytes in the center. As a result, increased platelet accumulation occurred downstream of the stent struts.

Coronary stent implantation is commonly performed to treat acute myocardial infarction and unstable angina. 30 55 56 57 58 These stent implantations often result in blood flow disturbance ( Fig. 3 ). The stent struts located at the vessel wall disturb blood flow locally. Platelets flowing to the center of the blood flow, owing to the presence of stent struts, are pushed back by the effects of centrally flowing heavy erythrocytes. This results in greater platelet accumulation distal to the stent strut. 4 59 The shape of the stent strut largely influenced the amount of platelet accumulation downstream of the stent strut. 60 Computer simulations of platelets confirmed the physical basis of stent-induced platelet accumulation. 61

**Fig. 4:**
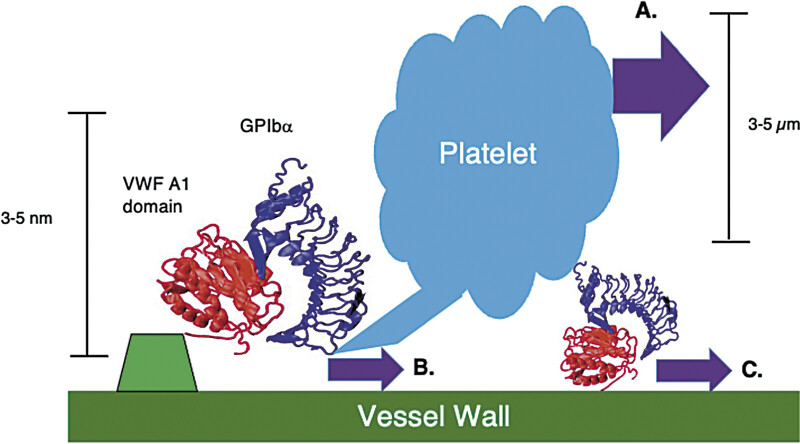
Balance of Forces Supporting Platelet Adhesion Mediated by GPIbα–Von Willebrand Factor (VWF) and the Hydrodynamic Detaching Force Generated by Blood Flow. Platelets adhering to the site of endothelial damage experience a fluid dynamic force that detaches them from the vessel wall. (Force A) Platelet adhesion is supported by the force generated by GPIbα binding to VWF. (Force B and Force C) Platelet adhesion persists as long as the integrated force of GPIbα–VWF is greater than that of the detaching force.

The role of blood flow in platelet adhesion differed substantially before and after platelet adhesion. Before platelet adhesion is completed, the major role of blood flow is to transport circulating platelets to the site of endothelial damage, 62 thus contributing to thrombus formation and growth. The direction of blood flow components toward the vessel wall is helpful for platelet adhesion. After platelet adhesion is complete, the major role of blood flow changes from enhancing platelet thrombus growth to detaching the platelets that have already adhered to the vessel wall. 63 64 Thus, the fluid dynamic force rather contributes to thrombus dissociation. 4 The integrated strength of the fluid dynamic force applied to the platelets should be equivalent to (or less than) the integrated strength of the binding force generated by the interaction of the adhesive protein with ligands. Fig. 4 is a simple model that supports platelets with two GPIbα molecules bound to VWF located at the vessel wall. Most likely, the fluid dynamic force applied to platelets changes over time because of the pulsatile nature of blood flow. However, the total force applied to the platelets (fluid dynamic force A) should be less than the adhesion force B plus C to keep the platelets bound to the vessel wall. The easiest way to increase the binding force at B and C after a sudden increase in force A by pulsatile flow is to increase the length of pseudopods B or C to increase the spring force. Biological experiments have revealed the presence of pseudopods that support platelet adhesion. 46 Moreover, both the number and length of pseudopods increased when the probability of GPIbα binding to VWF was blocked by an antibody against VWF. 46 The inhomogeneous distribution of GPIbα on platelets aids in pseudopod length-mediated force adjustment mechanisms. 1

The blood flow-dependent movements of the platelets were mathematically modeled as previously described. 61 65 The initial model treated platelets as particles that adhere to the site of vessel injury. The binding characteristics of the particles after initial adhesion were modeled to represent the results of the biological experiments. These simple platelet models reproduced the markedly greater platelet accumulation downstream of the stent struts 61 and the antiplatelet effects of GPIIb/IIIa and P2Y _12_ ADP receptor blockers ( Supplementary Fig. S1 , available in the online version). 66 Then the particle model of platelets was extended to a voxel simulator. 1 The expansion from a simple particle model to a voxel simulator enabled the representation of heterogeneous distributions of various platelet functional proteins, such as GPIbα. GPIbα was represented by a small number of particles located in the cell membrane. Once the particle was bound to the injured vessel wall, a pseudopod with spring elongation was formed to support platelet adhesion, as shown in Supplementary Fig. S2 (available in the online version). The developed voxel model divided single platelets into 20 × 20 × 40 voxels. This only represents a rough picture of platelet function constructed from regions with heterogeneous biological characteristics. Further development of a detailed model that separates the platelets into distinct regions representing the functions of various molecules is required.

## Physical Basis of Platelet Activation

Upon activation, platelets undergo substantial changes in their shape and biological functions. 67 68 69 70 71 Numerous biological events occur in the platelets upon activation. Of these, an increase in the intracellular calcium ion concentration ([Ca ^2+^ ] _*i*_ ) is noteworthy. 72 73 74 Intracellular calcium-dependent protein kinase mediates the phosphorylation of various intracellular proteins. 75 76 Platelet adhesion mediated by GPIbα binding with VWF exposed at the site of endothelial injury could also be expressed in a voxel simulator. 1 In this model, activation signals are generated locally around GPIbα molecules interacting with VWF. The spread velocities of the activation signals were determined based on the velocity of the intracellular spread of the increased [Ca ^2+^ ] _*i*_ . 72 Once the activation signal(s) reached the region of the platelet membrane, GPIIb/IIIa was activated. All platelets were activated when activation signals are spread throughout the platelet. Subsequently, other flowing platelets began interacting with the platelets that are stably bound at the site of endothelial damage. This simple particle simulator can be expanded to a cell scale using a voxel model. 1 These small changes in the intracellular proteins can provide a driving force for large structural and functional changes in the extracellular domains of membrane proteins. 77

The affinity of plasma ligand proteins to platelets (such as fibrinogen or VWF) increases substantially when platelets are activated. 78 79 80 These ligand proteins bind with platelet GPIIb/IIIa (integrin α _IIb_ β _3_ ) only when platelets are activated. These activation-dependent changes in the affinity of integrin α _IIb_ β _3_ is driven by the structural changes in their extracellular domain. 72 78 81 82 However, the physical basis of the activation-dependent structural changes in the extracellular domain of GPIIb/IIIa is still to be elucidated.

**Fig. 5:**
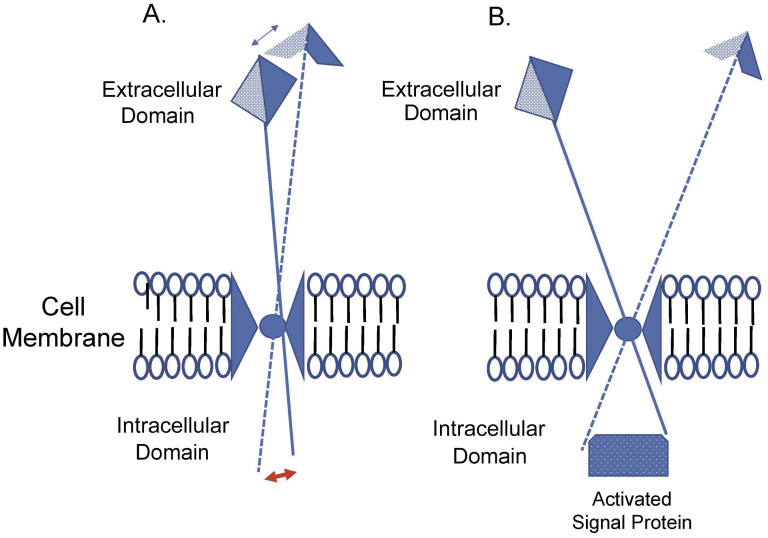
Molecular Leverage Mechanism Linking the Small Intracellular Structural Changes with Substantial Changes in the Extracellular Domain of GPIIb/IIIa. According to the molecular leverage hypothesis, the intracellular domain of GPIIb/IIIa is structurally flexible in non-activated platelets. ( **A** ) The rapid increase in intracellular calcium ion concentrations caused small structural changes in signaling proteins that could bind to the intracellular domain of GPIIb/IIIa. The structural flexibility of the intracellular domain of GP IIb/IIIa is reduced by binding to activated signal proteins. ( **B** ) Then the small structural changes in the intracellular domains of GPIIb/IIIa result in substantial structural changes in the extracellular domains of GPIIb/IIIa as a result of molecular leverage.

Biological experiments revealed that the intracellular environment of platelets changes substantially upon activation. 72 83 84 The “molecular leverage” hypothesis was proposed for linking physical events occurring inside to the outside of platelet cells. Based on this hypothesis, the mechanism of large structural changes in the extracellular domain of GP IIb/IIIa in response to small structural changes in their intracellular domains could potentially be explained 77 ( Fig. 5 ). The leverage is composed of the cellular membrane as the fulcrum of the lever. Small structural changes in the intracellular force point representing the intracellular domain of GP IIb/IIIa resulted in substantial structural changes in the extracellular force point representing the extracellular domain ( Fig. 5 ). The similar “molecular leverage” mechanism may mediate various functional changes in various cells not limited to platelets. The biological events that occur inside and outside platelets upon activation have been elucidated through various biological experiments. However, it should be noted that “molecular leverage” remains a hypothesis, and further quantitative modeling and validation experiments are required to validate this hypothesis as a true mechanism of platelet activation.

Another important functional change in platelets upon activation is an increase in their procoagulant activity. 85 86 87 88 In addition to microparticle release, the extracellular expression of negatively charged phospholipids such as phosphatidylserine (POPS) increases substantially with platelet activation. 89 90 91 92 Biological experiments revealed the increased POPS expression on activated platelets. 66 93 Yet, the mechanism is still to be elucidated. POPS expression is likely associated with an increase in intracellular calcium ion concentration. However, the link between increased [Ca ^2+^ ] _*i*_ and the extracellular expression of POPS is unknown. Biochemical experiments revealed the important role of transmembrane protein 16F (TMEM16F) as a phospholipid scramblase. 92 94

**Fig. 6:**
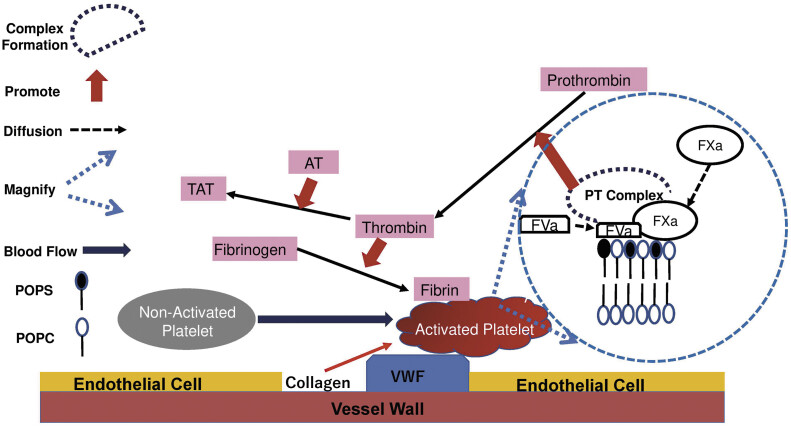
The Model Interplay Between Platelets and Coagulation System. Once non-activated platelets adhere to the von Willebrand factor (VWF) exposed at the site of endothelial damage, they are activated by interactions with VWF, collagen, and other soluble factors, such as ADP. Negatively charged phospholipids such as phosphatidylserine (POPS) are expressed on the outside of the activated platelet membranes, as shown in the magnified image in the blue circle. Various coagulation factors, such as activated factor X (FXa) and FVa, accumulate around the POPS to form the prothrombinase (PT) complex. Conversion from prothrombin to thrombin occurs efficiently in the PC complex to form fibrin around activated platelets. POPC, phosphatidylcholine; PT, prothrombinase; TAT, Thrombin antithrombin III complex.

Activated platelet-derived procoagulant activity was modeled as shown in Fig. 6 . The initial event is always endothelial disruption. Subsequently, non-activated platelets promptly adhered to the site of endothelial damage. They are activated by interactions between collagen and VWF. Negatively charged POPS phospholipids are expressed externally in the lipid bilayers of activated platelets. Subsequently, various coagulation factors, including those with Gla domains, such as activated factor X (FXa), 95 96 97 98 and those without Gla domains, such as coagulation factor V (FV), accumulate to form the prothrombinase (PT) complex. 90 99 100 101 Prothrombin can be converted efficiently to thrombin by the effects of PT complex. Thrombi then became larger by interacting with platelets and fibrin. This thrombus formation process can be mathematically simulated using a computer simulator. 102

The mechanism of pathological thrombus formation resulting in atherothrombotic events, such as myocardial infarction, can be understood qualitatively, starting from the functions of various proteins and cells. 30 However, there are substantial heterogeneities in the positions and functions of various proteins and cells that make it difficult to establish a simple “theory” for understanding the pathophysiology of various diseases from molecules or cells. Our aim for the next step is to construct pathophysiological events such as myocardial infarction by integrating simple physical equations to develop “theoretical medicine.” Biological cells and molecules are constructed from these atoms. The behavior of atoms depends on simple physical equations, such as Newton's laws of motion. Then the future directions of “theoretical medicine” may depend upon the directional changes from molecular biology to the new era of “atomic biology.” The progression of high-performance computers and information technology may help develop the basis of “atomic biology.” The concept is still highly speculative, and it remains uncertain how precisely we will be able to elucidate the pathophysiology of various diseases in the era of “atomic biology.” Moreover, it remains difficult to foresee how precisely we can predict the future onset of various diseases and the extent to which we can aid in their prevention.

## Physical Basis of Platelet Aggregation

**Fig. 7:**
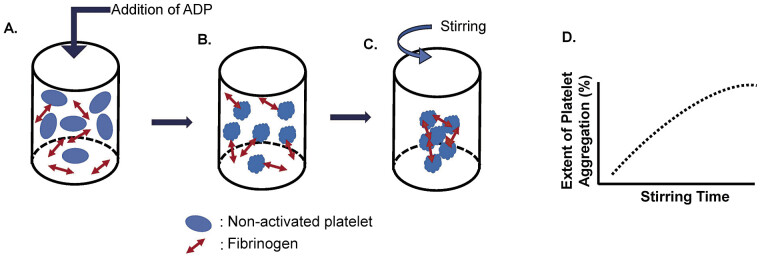
Platelet Aggregation. Platelets in platelet-rich plasma (PRP) are activated by the addition of soluble activating agents such as ADP. ( **A** ) Soluble fibrinogen (red arrow) binds to activated platelets, as shown in blue ( **B** ). Activated platelets form aggregates mediated by fibrinogen ( **C** ). The extent of platelet aggregation was calculated from the light transmittance of PRP ( **D** ).

The platelet function was assessed using various biological assays. Platelet aggregation is the most common target in clinical practice. 31 103 104 105 106 107 Typically, cloudy platelet-rich plasma (PRP) excluding erythrocytes is prepared. The addition of platelet-activating agents such as ADP, thrombin, or collagen activates platelets in PRP. Once platelets are activated, soluble fibrinogen binds to the activated platelets through their activated GPIIb/IIIa. 78 79 All soluble factors, including ADP, could reach the platelets by physical diffusion phenomena ( Fig. 7A ). Then external forces such as stirring cause fluid dynamic forces to move activated platelets to collide with each other to form aggregates ( Fig. 7C ). Sizes of platelets with a diameter of 2 to 5 µm are small but not small enough for physical diffusion in the PRP. The extent of platelet aggregation was measured as the light transmittance of the PRP. Light transmittance was low in the cloudy PRP. It increased when the number of particles in the PRP (platelets or platelet aggregates) decreased in the presence of platelet aggregates. The extent of platelet aggregation was calculated by measuring the transmitted light intensity using the Beer–Lambert equation. 107 In this equation, the log (L _t_ /L _i_ ) = −k × l × c where Lt is the transmitted (L _t_ ) and incident light intensity (L _i_ ), k is the absorption coefficient, l is the effective light pass, and c is the single platelet count. Using constant incident light, the extent of platelet aggregation was calculated. 107 108

Traditional agonist-induced platelet aggregation reflects the physical principles of diffusion of soluble substances and platelet movement in response to fluid forces and collisions. The extent of platelet aggregation was measured by absorption spectroscopy using the Beer–Lambert equation.

## Conclusion

Platelets play an important role in hemostasis and thrombus formation. Recent progress in computer technology and mathematical modeling has enabled the reconstruction of various biological functions of platelets based on simple physical principles. The validity of these mathematical models was confirmed through detailed quantitative biological experiments. The future progress of “theoretical medicine,” starting from simple physical principles to understanding complicated biomedical phenomena, is expected in platelet biology. Theoretically, it is important to address the enormous heterogeneity of biological phenomena that occur at scales such as molecules, cells, tissues/organs, and the human body.

## Notes

**What is known about this topic?**

- Biological and medical research revealed concrete biological mechanisms of various platelet functions.
- However, unravelling the complex mechanisms underlying various platelet functions has been difficult to integrate.

**What does this paper add?**

- Recent progress in high-performance computer and information technology enables to explain the complex pathophysiological mechanisms of platelet including the VWF–platelet interaction under blood flow conditions using the simple mechanism of physics.
- The future direction of constructive “theoretical medicine and biology” starting from atomic movements is expected to follow.
