# Supplementary material for: Physical and Mechanobiological Basis of Biological Functions of Platelets
Source: Thromb Haemost. 2025 Aug 20;126(7):661–72. doi: 10.1055/a-2676-4451 (PMC13282153; doi:10.1055/a-2676-4451)

Supplemental Fig. 1. Particle Model of Platelet Simulator

This figure comprises six videos. Videos A-1, A-2, B-1, and B-2 represent the behavior of the platelet particle model. Videos A-1 and A-2 represent three-dimensional views of platelet adhesion. Videos B-1 and B-2 represent the side views of the adhering platelets. Blue particles represent the non-activated platelets, and the red particles represent the activated platelets. Platelets were modeled to be activated when they adhered to the site of vessel damage. Details of the modeling have been published previously. [62] The simulation calculation was conducted with the parameters of vessel diameter of 40 μm, wall shear rate of 1,200 s^{-1}, platelet density of 1×10^6 /mm^3, and the length of endothelial injury of 10 μm. The non-activated platelets were allowed to transiently adhere to sites of endothelial damage. The adhesion settled and stabilized after platelet activation. (A-1 and B-1) Platelet accumulation did not occur substantially when an activation-dependent increase in platelet adhesion did not occur. (A-2 and B-2).

Biological experiments on human blood with platelets rendered fluorescent by the addition of mepacrine perfused with immobilized VWF are shown in Figure C-1. The results in the presence of the $P2Y_{12}$ receptor inhibitor AR-C69931MX are shown in C-2.

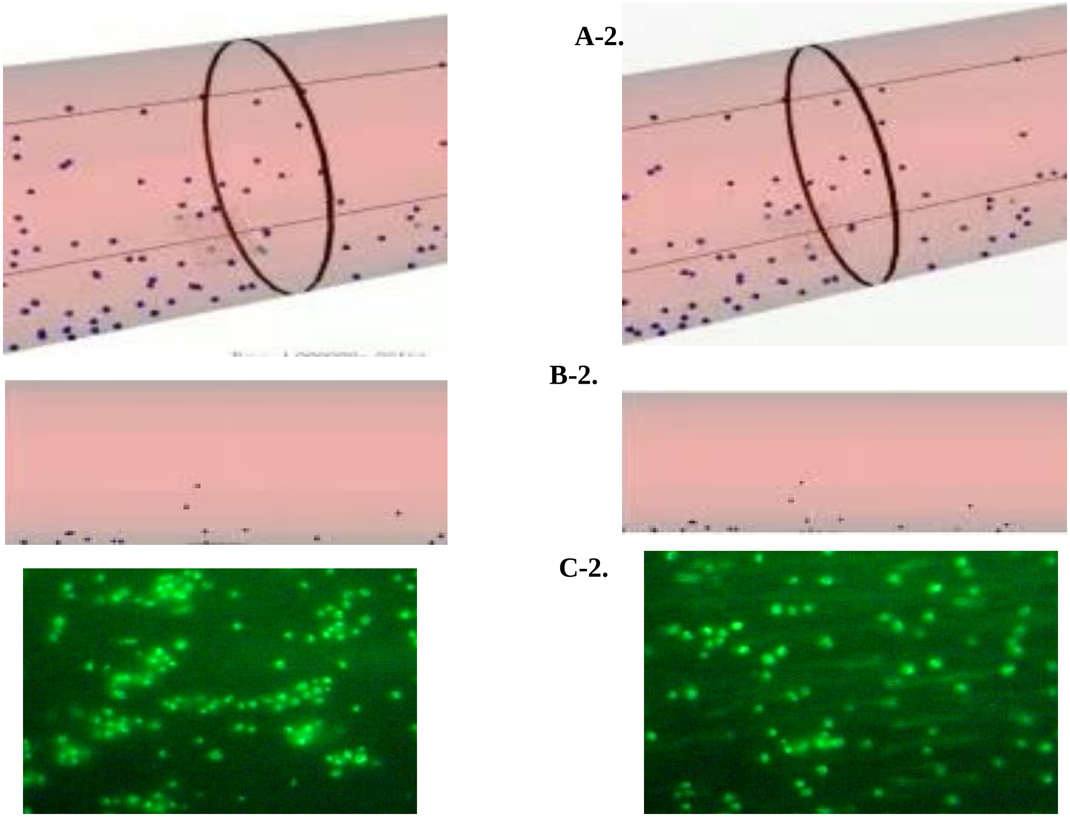

Supplemental Fig. 1

Supplemental Fig. 2. Pseudopod Model

The distribution of GPIb in platelet membranes was not homogeneous. [1] Platelets adhere to the site of endothelial damage in regions of the membrane where GPIb is abundant. (Panel A and Panel B) The body of platelets receives fluid dynamic force to detach them from the vessel wall. Subsequently, the adhesion force in this model increased with the elongation of the pseudopods. (Panel C)

The computer simulation model implementing the function of pseudopod elongation is shown in Movie D.

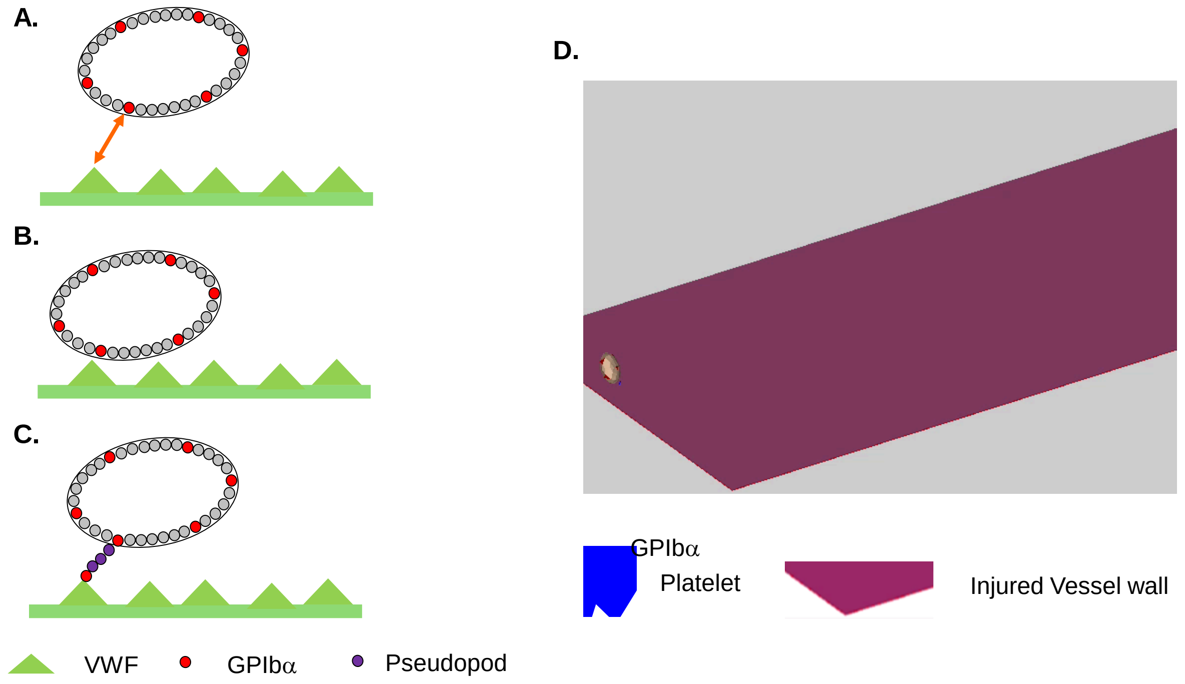

Supplement: Supplementary file 1 — Supplementary Material [file 10-1055-a-2676-4451_26872906.pdf]
